# When the SAINT goes marching in – A novel transcranial magnetic stimulation protocol shows miraculous promise

**DOI:** 10.1192/j.eurpsy.2023.1768

**Published:** 2023-07-19

**Authors:** J. D. Vieira de Andrade, M. J. Brito

**Affiliations:** Psychiatry Integrated Responsibility Centre, Coimbra Central University Hospital, Coimbra, Portugal

## Abstract

**Introduction:**

Transcranial Magnetic Stimulation (TMS) is a non-invasive neuromodulation tool with a growing body of clinical evidence demonstrating positive outcomes in patients with treatment-resistant depression (TRD) as sole or adjuvant therapy. Theta-burst stimulation (TBS), specifically intermittent TBS (iTBS), uses short intermittent pulse trains to cut each session’s duration to 10% of the original repetitive TMS protocol sessions, making it a more appealing option given that it shows similar efficacy. Nevertheless, the number of sessions required remains the same, with a single protocol lasting around 4 to 6 weeks, or longer. However, a new protocol has very recently been approved by the FDA for application in TRD, called the SAINT (Stanford Intelligent Accelerated Neuromodulation Therapy), which reduces treatment duration to 5 days.

**Objectives:**

To ascertain what evidence supports the SAINT protocol and its efficacy by reviewing available published literature.

**Methods:**

A PubMed database search was performed and the main findings of selected studies were summarized.

**Results:**

Three articles were found, which consisted of clinical trials with small study samples of TRD patients. One study found a 90% remission rate after the aforementioned 5-day treatment regimen, with another reporting a 79% response rate after a double-blinded trial. All studies reported no difference in tolerability compared with regular iTBS protocols.

**Conclusions:**

The SAINT protocol shows promising preliminary results, with efficacy, tolerability and safety of use comparable with that of TMS protocols already in use. The reduction in treatment duration that this intensive option is based on is a significant improvement for applicability in clinical practice, which might increase patient compliance and offer quicker results. Further studies are required to evaluate whether the remission rates are maintained in the long term.

**Disclosure of Interest:**

None Declared

